# Supplementary material for: Chirality control of a single carbene molecule by tip-induced van der Waals interactions
Source: Nat Commun. 2023 Jul 26;14:4500. doi: 10.1038/s41467-023-39870-y (PMC10371978; doi:10.1038/s41467-023-39870-y)
Supplement: Supplementary file 1 — Supplementary Information [file 41467_2023_39870_MOESM1_ESM.pdf]

Supplementary Information for

Chirality control of a single carbene molecule by tip-induced van der Waals interactions

*Yunjun Cao[1], Joel Mieres-Perez[2,3], Julien Frederic Rowen[4], Elsa Sanchez-Garcia[2,3], Wolfram Sander[4], and Karina Morgenstern[1]**

[1] Physical Chemistry I, Ruhr-Universität Bochum, Universitätsstr. 150, D-44801 Bochum, Germany

[2] Computational Bioengineering, Technical University Dortmund, Emil-Figge-Straße 66, 44227 Dortmund, Germany

[3] Computational Biochemistry, Universität Duisburg-Essen, Universitätsstr. 2, D-45141 Essen, Germany

[4] Organic Chemistry II, Ruhr-Universität Bochum, Universitätsstr. 150, D-44801 Bochum, Germany

*Corresponding author: karina.morgenstern@rub.de

Supplementary Note 1. Synthesis of DPDM

General: All chemicals were used as received without further purification. Aluminum oxide (neutral, Brockmann Activity I) was deactivated by addition of water to reach Brockmann Activity IV one day prior to its use as stationary material for column chromatography. NMR spectra were recorded on a Bruker Neo-400 MHz spectrometer and the chemical shifts are given in ppm referenced to DMSO-d_6 (^{1}H NMR: 2.5 ppm).

Synthesis:

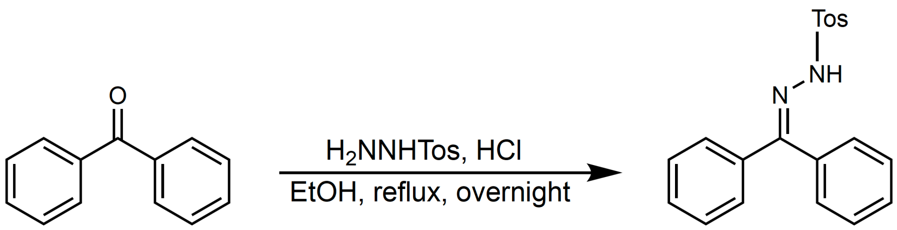

Following a literature procedure,[1] 2.0 g (11 mmol) of benzophenone (99%, Carl Roth) and 4.22 g (22.7 mmol) of *p*-toluenesulfonyl hydrazide (97%, Sigma-Aldrich) were suspended in 60 mL of ethanol (99.8 %, Sigma-Aldrich) and 1 mL of conc. HCl (37%, VWR) was added under stirring. The suspension was refluxed overnight and after cooling down the precipitate was filtrated and washed with small amounts of ethanol and dried *in vacuo*. Purification was achieved by recrystallization from ethyl acetate (HPLC grade, VWR). 2.37 g (6.8 mmol, 61.8 %) of benzophenone tosylhydrazone were obtained as colorless crystals.

^{1}H NMR (400 MHz, DMSO-d_6): δ/ppm = 10.41 (s, 1H), 7.83 (d, J = 8.1 Hz, 2H), 7.52 (dd, J = 5.0, 1.9 Hz, 3H), 7.43 (d, J = 8.1 Hz, 2H), 7.39 – 7.30 (m, 3H), 7.28 – 7.24 (m, 2H), 7.24 – 7.20 (m, 2H), 2.40 (s, 3H).

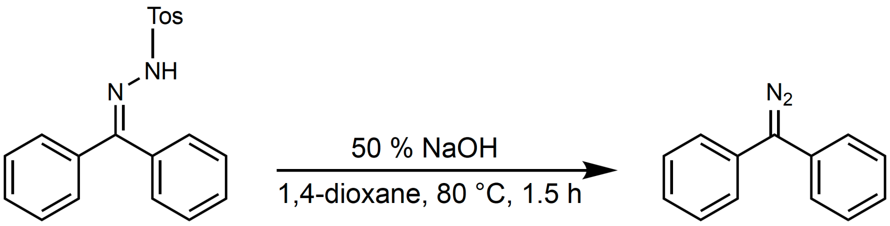

Following a literature procedure,[2] 2.0 g (5.7 mmol) of benzophenone tosylhydrazone were suspended in 25 mL of 1,4-dioxane (p.A., Fisher Scientific) and 3 mL of 50% aq. NaOH solution (NaOH pellets, Sigma-Aldrich) were added. The suspension was stirred vigorously at 80 °C for 1.5 hours. After cooling down to room temperature, 20 mL of distilled water were added, the organic phase separated, and the aqueous phase was extracted three times with 20 mL n-pentane (99%, VWR) until it was colorless. The combined organic phases were washed two times with 50 mL of distilled water, dried over $MgSO_4$ (99%, Carl Roth) and evaporated *in vacuo*. Purification was achieved by column chromatography (deactivated aluminum oxide (neutral, Acros Organics, Brockmann Activity IV); n-pentane as eluent). 390 mg (2.0 mmol, 35.2 %) of diphenyldiazomethane (DPDM) were obtained as a purple solid.

^{1}H NMR (400 MHz, DMSO-d_6): δ/ppm = 7.45 (dd, *J* = 8.3, 7.3 Hz, 4H), 7.30 (dd, *J* = 8.5, 1.2 Hz, 4H), 7.24 (t, *J* = 7.4 Hz, 2H).

Supplementary Discussion 1. Chirality change of DPC in gas phase

The diphenylcarbene (DPC) is helically chiral in gas phase. It exists as two enantiomeric states M and P. The two phenyl rings are rotated by a dihedral angle of ± 46° (labels 1, 2, 3, 4 of M and P, Supplementary Fig. 1a) to reduce steric hindrance.[3] To estimate the energy barrier of the chirality change, we calculate the potential energy by altering the dihedral angle of the DPC molecule in steps of 1° (Supplementary Fig. 1b). The calculated energy barrier to change the chirality of DPC by rotating its C–C–C bonds is 72 meV, going through a transition state (TS) where its two phenyl rings are coplanar (Supplementary Fig. 1b). The calculated chirality change in gas phase with a low energy barrier corroborates the flexible nature of the C–C–C bonds of DPC.

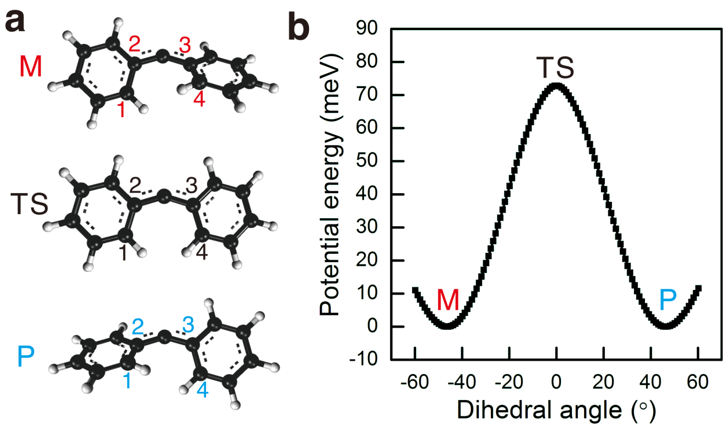

Supplementary Figure 1. Chirality change of DPC in gas phase. (a) Enantiomeric state of the DPC molecule in gas phase: M form, P form, and a transition state TS. Labels 1, 2, 3, and 4 of carbon atoms illustrate the dihedral angle of DPC. (b) Relaxed potential energy of DPC along the dihedral angle by steps of 1°. Structures of the DPC molecules in gas phase are optimized at the B3LYP-D3/def2-TZVP level of theory.

Supplementary Discussion 2. DPC enantiomers in different orientations.

In the main text, we show DPC enantiomers in two of their six orientations (Fig. 2c,d). Here, we display DPC enantiomers in their six orientations (Supplementary Fig. 2). The R-type and L-type enantiomers are rotated with respect to the <112> directions of the surface by angles of $\pm(16\pm2)°$, respectively.

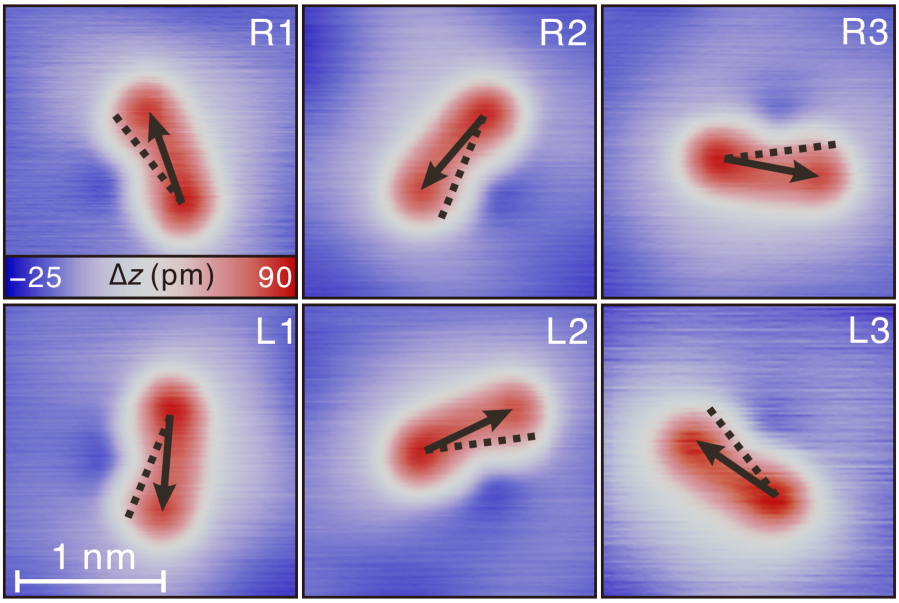

Supplementary Figure 2. DPC enantiomers in different orientations. R-type (upper panel) and L-type (lower panel) enantiomers on Cu(111). Arrows point from the brighter to the less bright protrusion along the main molecular axes. White dashed lines mark the <112> surface directions. Scanning parameters: V_b = 10 mV, I_t = 1 nA.

Supplementary Discussion 3. Immobile center during the chirality change of DPC

As discussed in the main text, the carbene center serves as an anchoring site on Cu(111). This leads to an immobile center during the chirality change of the DPC molecule. To determine this center, we plot the vertical bisectors (lines I and II, Supplementary Fig. 3a,b) of the main axes of the DPC molecules (solid lines, Supplementary Fig. 3a,b). The intersection of the two vertical bisectors determines the immobile center, i.e., the carbene center (grey spheres in Supplementary Fig. 3c,d). It should be pointed out that the bisection is based on the projection of the DPC molecules on Cu(111), which might introduce a small deviation in identifying the positions of the carbene center. Nevertheless, it corroborates the assignment in the main text that the carbene centers are situated at the sides of the depressions (Fig. 2c,d).

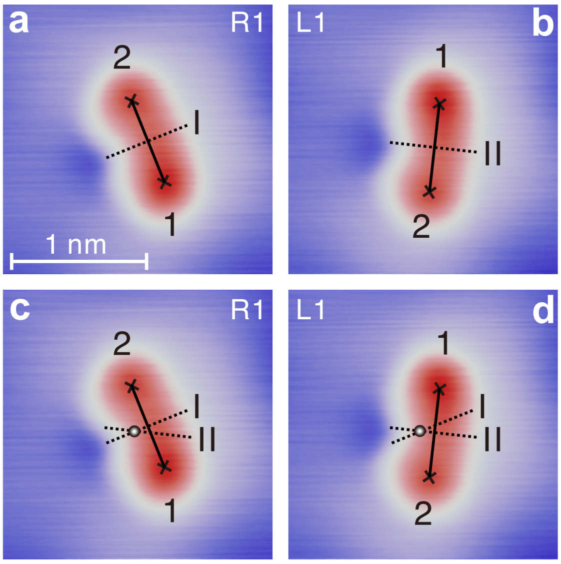

Supplementary Figure 3. Immobile center during the chirality change of DPC. (a,b) The bisection of (a) R1 and (b) L1 by vertical bisectors I and II (dashed lines). Crosses mark the centers of the protrusions (1 and 2) of R1 and L1. Solid lines connect 1 and 2 in (a) and (b), respectively. (c,d) Intersection of lines I and II from (a) and (b) marked by gray spheres. Scanning parameters: (a-d) V_b = 10 mV, I_t = 1 nA.

Supplementary Discussion 4. Schematics of chirality change of DPC on Cu(111)

In the main text, we flip the chirality of DPC by IET manipulation in pairs of R*n*↔L*n* (*n* = 1, 2, and 3, Fig. 2b and Supplementary Fig. 2). The calculations reveal that the higher phenyl ring (1) of DPC absorbs close to a bridge site of Cu(111) (Supplementary Fig. 4a,b), but the lower phenyl ring (2) to a hollow site (Supplementary Fig. 4c,d). The different adsorption sites lead to different ring-surface interactions and thereby different heights (h_1 and h_2) of the two rings with respect to the surface. Thus, the molecule is chiral on Cu(111). The strong interaction between the carbene center and the closest copper atoms of the surface (Figure 2h) is in agreement with that the carbene center is an immobile center during the chirality change (Supplementary Fig. 3). During IET manipulation, the injected electrons initiate the rotation of the two phenyl rings with respect to the carbene center, resulting in the change of their adsorption sites (Supplementary Fig. 4). It changes the relative heights of the two rings and thereby the chirality of DPC.

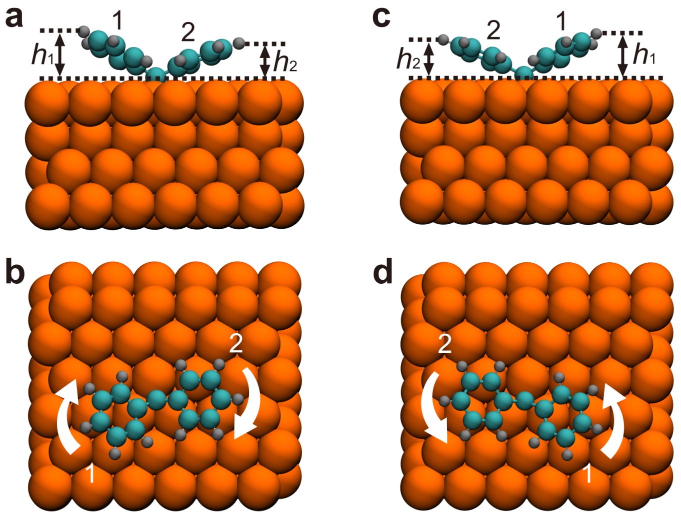

Supplementary Figure 4. Schematics of chirality change of DPC on Cu(111). (a-d) Optimized structure of R-type (a,b) and L-type (c,d) DPC on Cu(111). (a,c) Side view. (b,d) Top view. h_1 and h_2 mark the distances between the surface plane and the hydrogen atoms furthest away from the surface for each ring. 1 and 2 mark the two phenyl groups of DPC at different heights. The white arrows depict the rotation of phenyl rings with the change of their heights.

Supplementary Discussion 5. Time intervals during chirality change of DPC

The yield of the chirality change of the DPC molecule in the main text (Fig. 3c) is determined by a statistical analysis of the time intervals before the DPC molecule changes from the enantiomeric state under the tip to away from the tip. Exponential fits to the distributed probability give the time constants τ (Supplementary Fig. 5). At a voltage of 100 mV, the time constant is, at τ = (0.61 ± 0.06) s, more than three times larger than at 150 mV ((0.18 ± 0.01) s). The yield of the chirality change per electron is calculated from τ by $Y = 1/N = e/(I \cdot \tau)$ (Fig. 3c), where N is the number of electrons flowing through the DPC molecule per chirality change event, $e = 1.6 \times 10^{-19}$ C is the elementary charge, and I is the tunneling current.

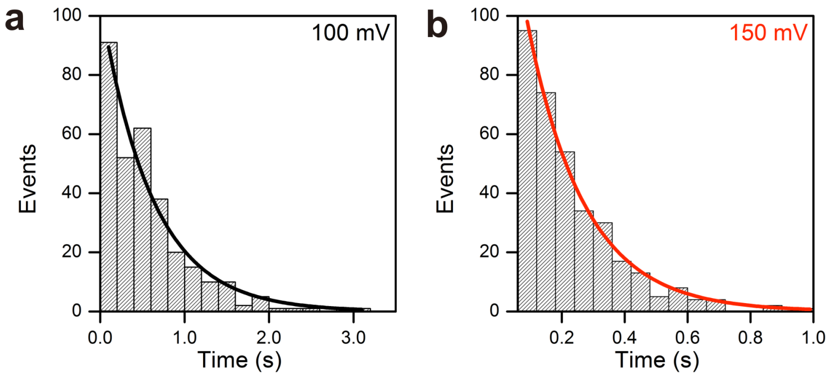

Supplementary Figure 5. Time intervals during chirality change of DPC. (a-b) Histograms of the time intervals before the DPC molecule changes from the enantiomeric state under the tip to away from the tip at fixed voltages of (a) 100 mV and (b) 150 mV. The setpoint current is 2 nA in (a) and (b). The solid lines (a) black and (b) red represent exponential fits $N(t) = N_0 e^{-t/\tau}$, where $N(t)$ is the quantity of events at time t, N_0 is the initial quantity of events, and τ is the time constant. The fitted time constants are (a) τ = (0.61 ± 0.06) s and (b) τ = (0.18 ± 0.01) s, respectively.

Supplementary Discussion 6. Determination of voltage threshold between two yield levels

In Fig. 3c of the main text, the electron yield Y vs. voltage V was fitted separately in three voltage ranges by two-level Boltzmann functions

$$Y = Y_{\mathrm{low}} + \left(Y_{\mathrm{high}} - Y_{\mathrm{low}}\right)/\left(1 + e^{-(V - V_{1/2})\cdot b}\right) \tag{1}$$

where Y_{low} and Y_{high} are the low and high yield levels in the corresponding voltage ranges, b is the apparent first-order constant, and $V_{1/2}$ is the voltage required to reach half the yield between Y_{low} and Y_{high}. Here, we demonstrate the fitting procedure in the range between 140 mV and 200 mV. As shown in Supplementary Fig. 6, the fitting curve (yellow) fits the electron yield Y (black squares) well. After having defined the two levels $Y_{\mathrm{low}} = 0.35 \times 10^{-9}$ per electron and $Y_{\mathrm{high}} = 0.79 \times 10^{-9}$ per electron, the fitted threshold (blue sphere) is given by the voltage $V_{1/2}$ at which

$$Y_{1/2} = (Y_{\mathrm{low}} + Y_{\mathrm{high}})/2 \tag{2}$$

Here, $V_{1/2} = (161 \pm 1)$ mV at $Y_{1/2} = 0.57 \times 10^{-9}$ per electron.

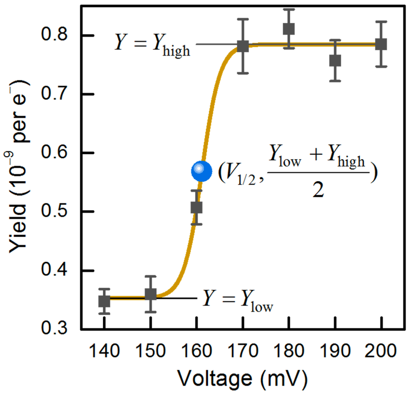

Supplementary Figure 6. Determination of voltage threshold between two yield levels: Chirality change yield Y per electron vs. bias voltage V. Black squares: experimental data; yellow line: result of the fitting; black lines: two fitted levels Y_{low} and Y_{high}; blue sphere: the middle point of $Y_{1/2} = (Y_{\mathrm{low}} + Y_{\mathrm{high}})/2$. The error bars of yield Y were derived from the errors of the time constants τ, which are obtained by fitting hundreds of events for each data point (for details see Supplementary Fig. 5).

Supplementary Discussion 7. *I-V* curve

In Fig. 3 of the main text, we concentrated on the chirality change of DPC at positive voltages, where a vibrational heating mechanism is deduced from the match of threshold voltage values to the energies of molecular vibrations. *I–V* curves support this conclusion. In Supplementary Fig. 7, we ramped the bias voltage above the DPC molecule from positive to negative voltage. Regardless of polarity, the *I–V* curve (black) fluctuates when the absolute value of the voltage surpasses the same threshold of ≈ 60 mV (gray background). Such fluctuations reflect the chirality change of the molecule, which is not observed in *I–V* curves recorded of the Cu(111) surface (pink line). The polarity-independent threshold for fluctuations in the *I–V* curve corroborates that the chirality change of DPC is induced by vibrational heating, a process which is triggered by inelastic electrons.

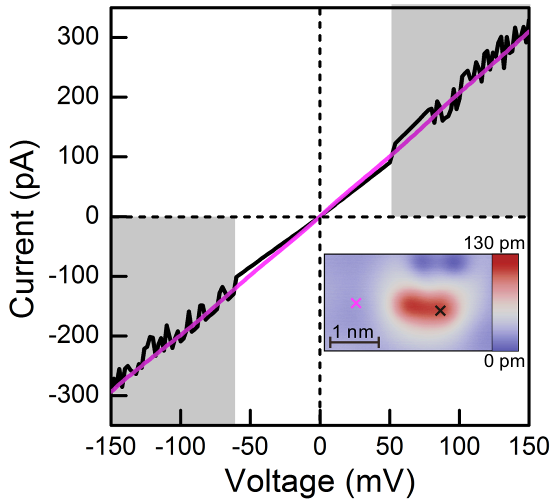

Supplementary Figure 7. *I-V* curve recorded at the crosses in the STM image in corresponding colors. The bias was ramped at a step size of 2 mV and a dwelling time of 2 s for each step. Gray background marks range of fluctuations above the molecule (black line). Inset: STM image of a DPC molecule recorded at scanning parameters of V_b = 10 mV, I_t = 10 pA.

Supplementary Discussion 8. Energy barrier of chirality change of DPC on Cu(111)

As discussed in the main text, the yield changes at three threshold voltages of (64 ± 2) mV (I), (125 ± 2) mV (II), and (161± 1) mV (III) (Fig. 3c). These values fit nicely to the infrared spectra of DPC in rare gas matrices, with one skeletal vibrational mode at 62 meV and two C-H deformation modes at 126 meV and 172 meV.[4] Power law fittings $\tau^{-1} \propto I^N$ give $N \approx 1$ (Fig. 3d), indicating a single tunneling electron being sufficient to excite the vibrational mode above the energy barrier of the chirality change (Supplementary Fig. 8). We thus propose that the energy barrier of the chirality change of DPC on Cu(111) is below the lowest threshold energy at (64 ± 2) meV (I).

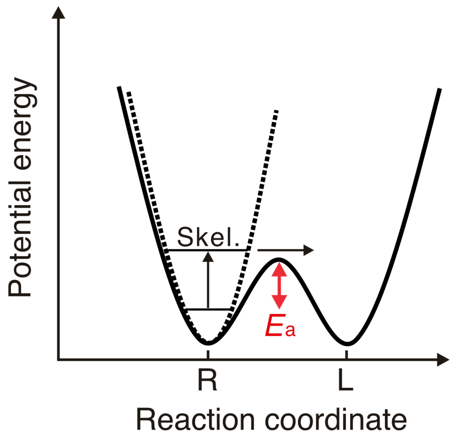

Supplementary Figure 8. Energy barrier of chirality change of DPC on Cu(111). Schematic model for the chirality change of DPC between its two enantiomers (R and L) by inelastic electrons. The excitation among the levels represents the vibrational transition (skeletal mode) to trigger the chirality change.

Supplementary Discussion 9. Estimation of *z*-offset

The observation of vacuum tunneling was established by the exponential dependence of the tunneling current on the width of the tunneling gap.[5] Based on this, we use an *I–z* curve to estimate the *z*-offset of the STM tip with respect to the initial setpoint at a tunneling resistance of 2 GΩ. A low voltage of 10 mV is applied during approach to avoid the chirality change of the DPC molecule. As expected, the tunneling current decays exponentially as a function of the *z*-offset (Supplementary Fig. 9). Based on the *I–z* curve, we calculate the *z*-offset of the STM tip from the corresponding tunneling parameters.

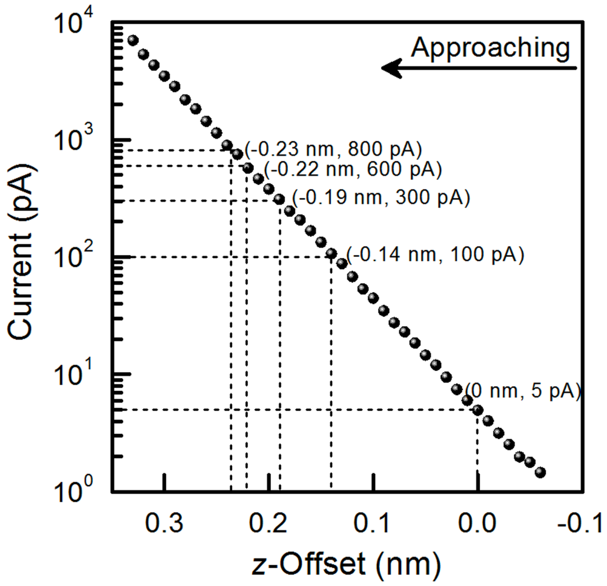

Supplementary Figure 9. Estimation of *z*-offset. Tunneling current as a function of *z*-offset (*I–z* curve) for the STM tip approaching one of the phenyl rings of DPC at a low voltage of 10 mV. The *z*-offset at 0 nm is the initial set point of IET manipulation (tunneling current of 5 pA, tunneling resistance of 2 GΩ). Typical points for IET manipulation are marked in the graph with *z*-offsets and corresponding tunneling currents.

Supplementary Discussion 10. Derivation of potential well depths from *I-t* trace

In Fig. 4 of the main text, we show the change of DPC between its two enantiomers with the *I–t* trace, the occupations in time, the normalized occupations, and potential well depths. Here, we exemplify at one data set (*z*-offsets of 0.14 nm, Fig. 4e-h) how to derive the potential well depths from the *I–t* trace via the occupations in time and the normalized occupations (Supplementary Fig. 10).

Step I: The occupations in time, $\mathrm{Occ_H}$ and $\mathrm{Occ_L}$, are derived from a histogram of the *I–t* trace, displaying two well-separated maxima at the current values I_H and I_L. Their relative areas yield the percentages $\mathrm{Occ_H}$ and $\mathrm{Occ_L}$ during which the molecule is either in its high state (H) or its low state (L).

Step II: The occupations in time must be normalized to occupations per electron because, given the same time interval, there are more electrons flowing through the molecule for a higher current value than a lower one. Thus, more switching events will be generated per time interval for a higher current value than a lower one. To compensate for this effect, we define a normalized occupation probability

$$P_i = \frac{\mathrm{Occ}_i \cdot I_i}{\mathrm{Occ_H} \cdot I_\mathrm{H} + \mathrm{Occ_L} \cdot I_\mathrm{L}} \qquad (3)$$

where I_i with i = H or L represent the high-current (H) and low-current (L) states. They reflect the probabilities per electron for the molecule staying in the H or L states.

Step III: The molecule switching between the two states is considered as a classic system following the Boltzmann distribution. Thus, the energy difference is extracted based on the Boltzmann distribution

$$P_\mathrm{H}/P_\mathrm{L} = e^{-\frac{\Delta E}{\mathrm{k}T}} \qquad (4)$$

where $\Delta E = E_\mathrm{H} - E_\mathrm{L}$ is the energy difference between the two minima of the double-well potential with depths of E_H and E_L, k is the Boltzmann constant, and T is the surface temperature. For the example in Supplementary Fig. 10, $\Delta E = 0.5$ meV is calculated from $P_\mathrm{H} : P_\mathrm{L} = 74.2\% : 25.8\%$ at $T = 5.1$ K.

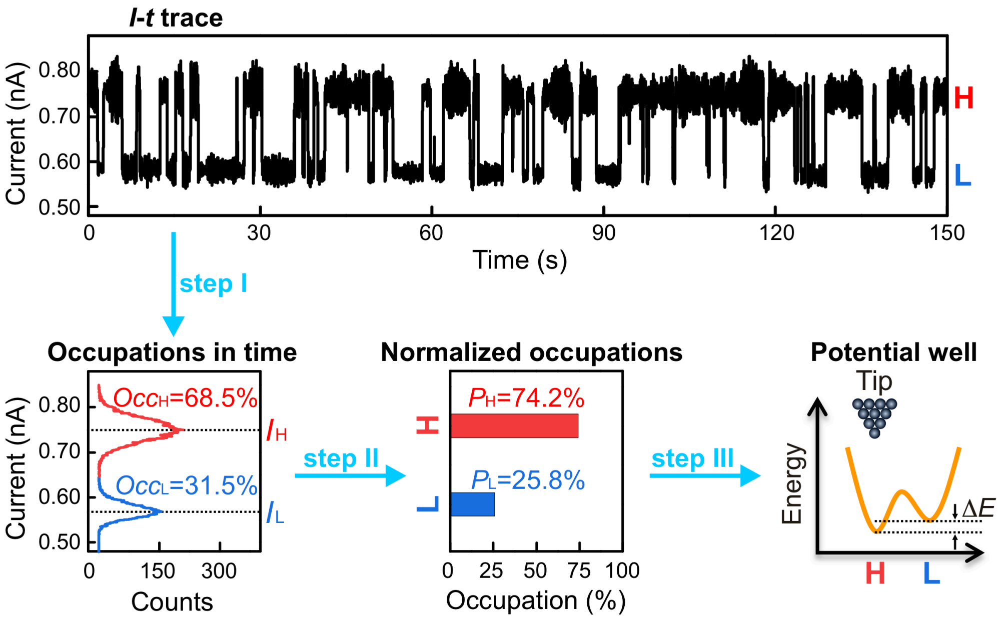

Supplementary Figure 10. Procedure to derive potential well depths from *I-t* trace. Scheme of the three steps: Step I: from the *I–t* trace to the occupation in time; Step II: from the occupations in time to the normalized occupations; Step III: from the normalized occupations to the potential well depths. Note that ΔE is largely exaggerated with respect to the energy barrier for chirality change because of more than an order of magnitude difference in energies.

Supplementary Discussion 11. Tip-induced enantiomeric excess

In the main text, we present the asymmetric distribution of DPC between its two enantiomers induced by an STM tip apex (tip #1). The normalized occupation P_H increases monotonously with decreasing tip-molecule distance (Fig. 4). Here, we show another example recorded by another STM tip apex (tip #2). Likewise, the molecule prefers to stay more and more in the high current state with decreasing tip-molecule distance (see *I*–*t* traces in Supplementary Figure 11a,d,g,j). The corresponding normalized occupation P_H increases from 52.5% to 96.8% (Supplementary Figure 11c,f,i,l). P_H slightly varies from tip to tip as expected for different shapes of the tip apexes, for example, it is 89.8% for tip #1 at a *z*-offset of 0.22 nm but 96.8 % for tip #2 at a smaller *z*-offset of 0.21 nm. The slight variations corroborate further the role of the vdW interactions between the tip apex and the molecule.

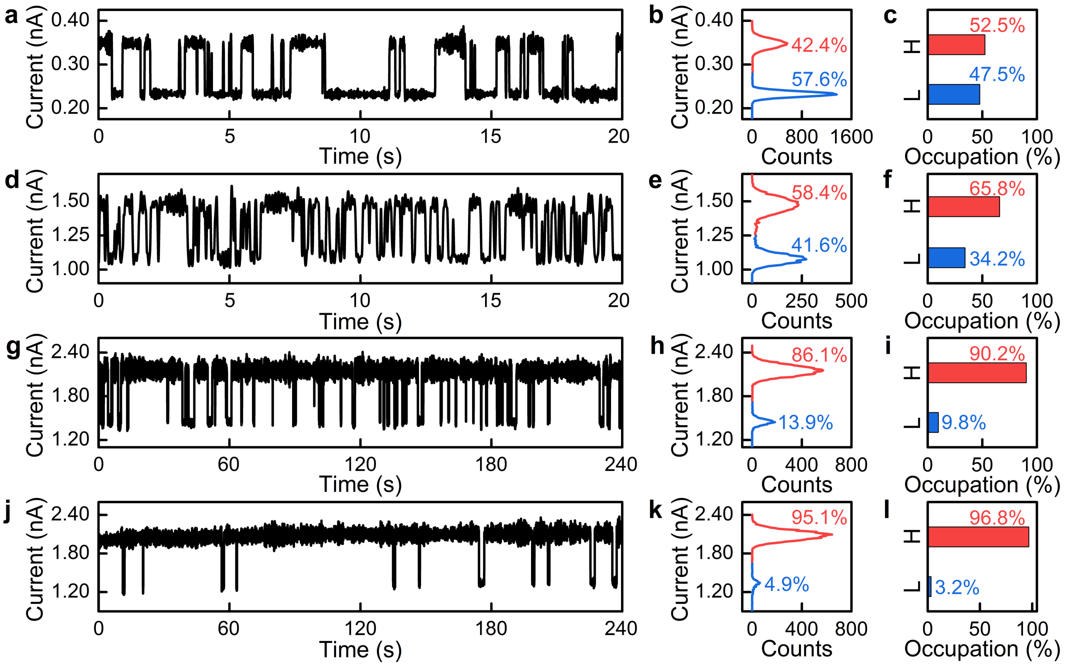

Supplementary Figure 11. Tip-induced enantiomeric excess. IET manipulation on one of the phenyl rings of DPC at *z*-offsets of 0.07 nm (a-c), 0.14 nm (d-f), 0.20 nm (g-i), and 0.21 nm (j-l) from an initial setpoint at a tunneling resistance of 2 GΩ. From left to right: (a,d,g,j) *I*–*t* traces, (b,e,h,k) current histograms (percentages in panels mark occupations in time for the high-current (red) and low-current (blue) states), (c,f,i,l) normalized occupations. Setpoint voltages: (a,b) 150 mV, (c) 50 mV, (d) 40 mV.

Supplementary Discussion 12. Influence of tip field on normalized occupation P_H

In the main text (Fig. 4), we demonstrate that the ratio of $P_H : P_L$ is largely altered by the tip-molecule distance. Here, we modulate the electric field at a fixed tip-molecule distance (*z*-offset of 0.08 nm) for excluding other causes than the vdW interactions for the alteration. Increasing the voltage by a factor of four influences the normalized occupation P_H only marginally (Supplementary Fig. 12). It excludes that the electric field induces the asymmetric distribution of DPC between its two enantiomers at closer tip-molecule distances.

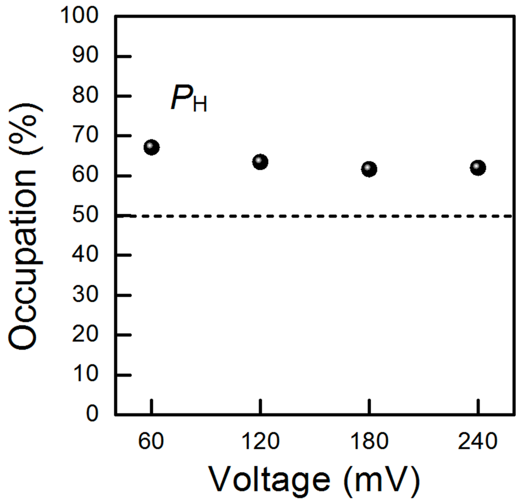

Supplementary Figure 12. Influence of tip field on normalized occupation P_H. Normalized occupation P_H of the DPC molecule versus the voltage at a fixed tip-molecule distance (*z*-offset of 0.08 nm).

Supplementary Discussion 13. Manipulating DPC by tip-induced interactions only

In the main text, we used inelastic electrons and tip-induced vdW interactions to mimic a chiral induction in a molecular assembly, where the chirality of the DPC molecule was reversibly switched in a well-controlled fashion without any unintended side processes (Fig. 1a). Here, we demonstrate the chirality change of DPC driven by tip-induced interactions only (Supplementary Fig. 13). However, this change demands stronger tip-sample interactions that not only change the chirality of the molecule but also induce unintended side-processes, such as rotation (Supplementary Fig. 13a to 13e) and translation (Supplementary Fig. 13g to 13i). Because more than one process is induced by the manipulation, the frequency shift (Δf) curves are often undefined. For instance, many Δf curves do not follow the excepted Lennard-Jones potential but are dominated by sudden jumps. Nevertheless, we present a relatively well-defined Δf curve which is altered by only one change to the molecule in the repulsive interaction regime (red arrow, Supplementary Fig. 13f). However, such changes have also been observed in the attractive interaction regime. Overall, these side processes undermine characterizing the influence of tip-induced interactions on the chirality change of the molecule by dynamic AFM.

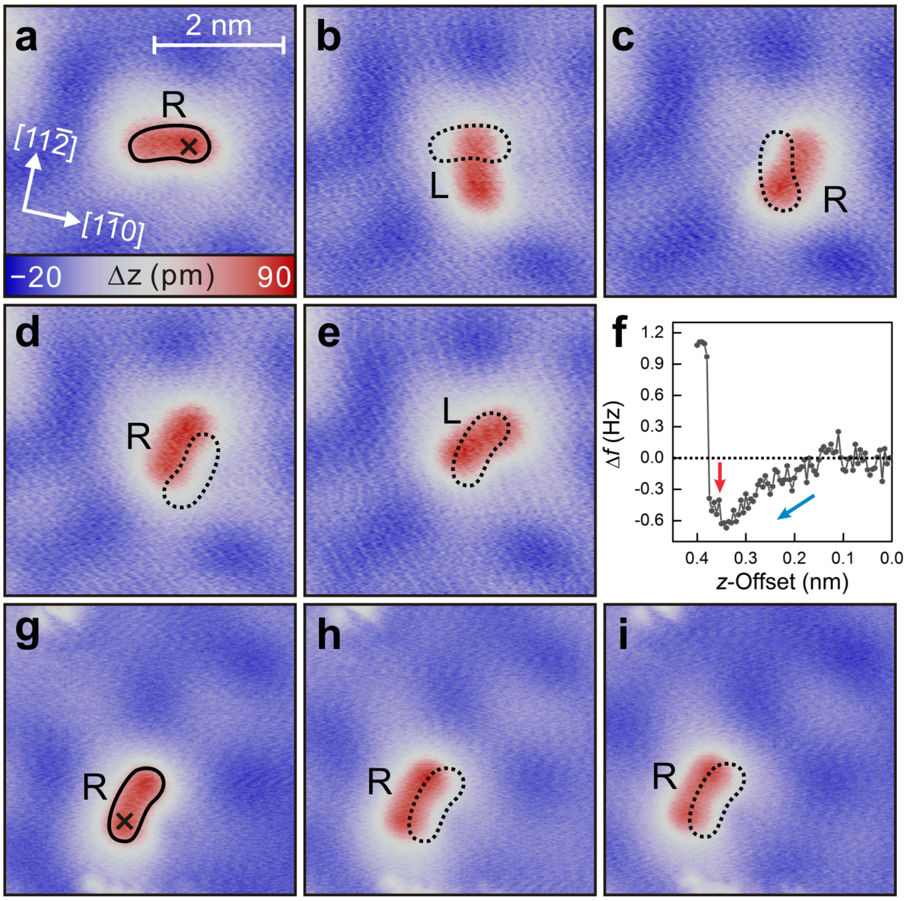

Supplementary Figure 13. Manipulating DPC by tip-induced interactions only. (a-e, g-i) STM images of sequential manipulation of two DPC molecules ((a-e): I, (g-i): II) by tip-induced interactions. The manipulation sites in (a-d) are the brighter protrusions and in (g,h) the less bright protrusions, as marked by crosses in (a) and (g), respectively. Contours (solid) in (a) and (g) mark the initial positions of DPC; contours (dotted) in (b-e) and (h,i) mark the positions of DPC in the previous frame. Scanning parameters: V_b = 10 mV, I_t = 10 pA. (f) Spectra of frequency shift Δf as a function of *z*-offset measured during the manipulation from (a) to (b). The *z*-offset is defined with respect to an initial setpoint of V_b = 10 mV and I_t = 10 pA. The blue arrow marks the approaching direction. The red arrow marks a discrete jump of Δf during the manipulation.

Supplementary References

1. Feng, X. W. *et al.* Copper-catalyzed nitrogen loss of sulfonylhydrazones: a reductive strategy for the synthesis of sulfones from carbonyl compounds. *Org. Lett.* **12**, 4408-4411 (2010).
2. Jonczyk, A. & Wlostowska, J. A Simple Method for Generation of Diazocompounds in an Aqueous Two-Phase System. *Synth. Commun.* **8**, 569-572 (1978).
3. Henzl, J., Mehlhorn, M., Gawronski, H., Rieder, K. H. & Morgenstern, K. Reversible cis-trans isomerization of a single azobenzene molecule. *Angew. Chem. Int. Ed.* **45**, 603-606 (2006).
4. Costa, P., Fernandez-Oliva, M., Sanchez-Garcia, E. & Sander, W. The highly reactive benzhydryl cation isolated and stabilized in water ice. *J. Am. Chem. Soc.* **136**, 15625-15630 (2014).
5. Binnig, G., Rohrer, H., Gerber, C. & Weibel, E. Tunneling through a controllable vacuum gap. *Appl. Phys. Lett.* **40**, 178-180 (1982).
